# Role of Starch Accumulation at the Mature Green Stage in Shaping Tomato Fruit Quality

**DOI:** 10.3390/plants15091364

**Published:** 2026-04-29

**Authors:** Hongxue Li, Heng Wang, Weijie Jiang, Yang Li, Mengjiao Liu, Hongjun Yu, Qiang Li

**Affiliations:** 1 College of Plant Science and Technology, Beijing University of Agriculture, Beijing 102200, China; 2 State Key Laboratory of Vegetable Biobreeding, Institute of Vegetables and Flowers, Chinese Academy of Agricultural Sciences, Beijing 100080, China; 3 College of Horticulture, Xinjiang Agricultural University, Urumqi 830052, China

**Keywords:** tomato, mature green fruit, starch content, total soluble solids, fruit quality

## Abstract

Tomato (*Solanum lycopersicum*) is a globally important vegetable crop, with fruit quality being a major focus of research. Starch serves as the primary carbohydrate reserve during early fruit development and functions as a key carbon precursor for flavor compound biosynthesis in later stages. To elucidate the role of starch accumulation in determining ripe fruit quality, we analyzed the relationship between starch content in mature green fruits and flavor-related traits across eight tomato cultivars. The results demonstrated that starch content at the mature green stage showed a significantly positive correlation with total soluble solids (TSS) content (r = 0.922) and a significantly positive correlation with total acidity content (r = 0.783) in red-ripe fruits. Furthermore, the expression levels of starch synthesis gene *AGPS1* and degradation gene *PWD* at the mature green stage were both significantly positively correlated with the final fruit TSS levels. These findings highlight the important role of starch accumulation during the mature green stage in shaping final fruit quality, providing a theoretical basis for breeding high-quality tomato varieties.

## 1. Introduction

Tomato (*Solanum lycopersicum* L.) is a globally significant vegetable crop, valued for its economic significance and nutritional benefits [1]. According to estimates from the Food and Agriculture Organization (FAO), global tomato production exceeded 188 million tons in 2024 highlighting its central role in global agriculture and food systems [2]. With advancements in modern agriculture, the demand for high-quality tomato has increased substantially [3]. The foundation of fruit quality is largely related to the internal carbohydrate metabolism of the fruit, particularly the dynamic changes in starch and sugars [4].

During the development of tomato fruits, the accumulation and degradation of starch play a pivotal role in final fruit quality [5]. The developmental process of tomato fruit can be divided into several major stages: fruit set, expansion, mature green, breaker, and red-ripe [6]. At the fruit set stage, rapid cell division occurs, accompanied by the initiation of starch biosynthesis [7]. Starch accumulation intensifies during the expansion stage, culminating at the mature green stage to provide essential carbon and energy reserves for the subsequent ripening phase [5,8,9]. Notably, the transition from the mature green stage to the breaker stage marks a metabolic turning point, during which starch undergoes rapid degradation into soluble sugars—mediated by hydrolytic enzymes such as amylases and debranching enzymes [10]. This efficient transformation of starch to sugars directly determines the sweetness and flavor of the fruit at the red-ripe stage [11].

Total soluble solids (TSS) represent a key indicator for evaluating tomato fruit quality [12]. TSS primarily consists of soluble sugars (such as fructose and glucose) and organic acids (such as citric acid and malic acid) [12,13]. TSS level as well as the ratio of soluble sugars to organic acids directly influence the fruit flavor and nutritional value [14]. Higher TSS content is generally associated with enhanced flavor intensity and firmer texture, traits that increase consumer preference and marketability [12]. As a result, TSS serves not only as a critical quality benchmark but also as a widely adopted selection criterion in tomato breeding programs [15]. Understanding the dynamics of TSS accumulation in different tomato cultivars, particularly in relation to starch metabolism, is therefore essential for developing strategies to improve fruit quality [16].

The relationship between starch metabolism and TSS accumulation is critical during tomato fruit development [17]. However, the underlying mechanisms remain limited. Therefore, in-depth research on the relationship between starch accumulation and TSS content during fruit development in different tomato cultivars will not only provide a theoretical basis for improving tomato fruit quality but also offer new insights into revealing the molecular mechanisms of carbohydrate metabolism in fruits.

In this study, we employed eight tomato cultivars to perform correlation analysis between starch in mature green fruits and the key quality metabolites (e.g., TSS, total acidity, vitamin C, lycopene, and free amino acids) in red-ripe fruits. Furthermore, we analyzed the expression of key genes involved in starch synthesis and metabolism in mature green fruits and examined their correlation with starch content. This study aims to elucidate the dynamic patterns of carbohydrate metabolism during tomato fruit development and provide a scientific basis for future quality improvement and optimized breeding strategies.

## 2. Materials and Methods

### 2.1. Plant Material

Eight tomato (*Solanum lycopersicum* L.) cultivars with distinct flavor profiles were selected: ‘Provence’, ‘Sai Xishi’ (SXS), ‘Baoshijie 913’ (BJ913), ‘Tiefan 807’ (TF807), ‘Yuanwei No.1’ (YW1), ‘Fentao No.1’ (FT1), ‘Xiangfei No.3’ (XF3), and ‘Busan 88’. These cultivars were selected according to their previously observed wide variation in fruit quality traits (Supplement Tables S2–S4).

### 2.2. Growth Conditions

The experimental plant materials were sown on 20 January 2024, and grown in seedling trays. On February 28, they were uniformly transplanted into a sunlight greenhouse at the experimental base of the Chinese Academy of Agricultural Sciences in Nankou Town, Changping District, Beijing. A randomized block design was adopted with three replicates, each consisting of eight plants. Border rows were established around the experimental plots. All plants were managed under uniform conditions. The cultivation substrate was coconut coir, and irrigation was carried out using a modified “Hongland” nutrient solution. Plants were trained to a single stem, with five fruit clusters retained. Each cluster was kept with 4 to 5 fruits, and the growing point was pinched off after leaving two leaves above the top cluster.

### 2.3. Test Methods

#### 2.3.1. Fruit Sampling

For each of the eight tomato cultivars and each growing season, three independent biological replicates were performed. Each biological replicate consisted of three randomly selected plants from the eight plants per replicate. At the mature green stage and red-ripe stage, three fruits per plant were harvested from the middle of the fruit cluster (the second to fourth clusters). The three fruits from the three plants (i.e., nine fruits per biological replicate) were pooled and thoroughly mixed. Subsamples of this pooled sample were used for the following determinations. All measurements were performed in technical triplicate, and the average value was taken as the result for that biological replicate.

#### 2.3.2. Soluble Solid and Acid Determination

At the time of harvest, tomato fruits were collected at the red-ripe stage. For each biological replicate, three fruits (one from each of three randomly selected plants within that replicate) were combined and homogenized. Total soluble solids content and acid content were determined using a handheld refractometer (Atago PAL-1, ATAGO, Tokyo, Japan). The refractometer was calibrated to zero with distilled water. Juice extracted from the fruits was then placed on the measuring surface of the refractometer for reading, and the obtained value represented the total soluble solids content and acid content of the fruits. For each genotype, three biological replicates were measured, and the average of these three values was reported as the total soluble solids content and acid content for that variety. At the same time, tomato mesocarp and exocarp were taken and flash-frozen in liquid nitrogen and stored at −80 °C for the next experiment.

#### 2.3.3. Soluble Sugar and Starch Determination

For each biological replicate, three fruits were pooled, and the pericarp was sampled after removing the seeds and placenta. The pooled pericarp tissue was flash-frozen in liquid nitrogen and ground into powder. The concentrations of glucose, fructose, and sucrose were determined using ultra-performance liquid chromatography coupled with a refractive index detector (UPLC-RID, Waters Corporation, Milford, MA, USA). Approximately 100 mg of the ground frozen fresh pericarp samples were homogenized and extracted in 1900 μL of water in a water bath at 80 °C for 30 min. After sonication (80 W for 10 min) and centrifugation (12,000 rpm for 10 min), the solution was filtered using a 0.22 µm polyethersulfone ultrafiltration membrane as the extraction solution. Subsequently, the extraction solution was mixed with equal acetonitrile for analysis. The UPLC analyses used an ACQUITY UPLC BEH Amide 1.7 μm as the analytical column (2.1 mm × 100 mm; Waters Corporation, Milford, MA, USA). In the mobile phase, acetonitrile was utilized as solvent A and 1 mg/mL ammonium hydroxide as solvent B. The temperature of the column and autosampler was 60 °C and 10 °C, respectively. Initially, the gradient of solvent B ran at 10% and used a flow rate of 0.2 mL/min for 2 min; subsequently, each soluble sugar was separated by linearly increasing the concentration of solvent B from 10% to 20% over 6 min, followed by washing with 10% solvent B for 2 min. The column was re-equilibrated for 5 min in the initial conditions described above. The mass spectrometer was operated in the multiple reaction monitoring (MRM) mode to detect the selective fragments of the [M-H]-precursor ions. Data analysis was carried out using MassLynx ver4.1 (Waters Corporation, Milford, MA, USA). The total soluble sugar concentration was estimated by summing the concentrations of sucrose, glucose, and fructose.

The remaining pellet obtained after extracting the soluble sugars was utilized to determine the starch concentration. Briefly, the supernatants containing the soluble sugar were discarded. The residues were then washed twice with water; after each wash, they were centrifuged for 10 min and the supernatant was removed. Subsequently, the remaining pellet was completely dried. The residues were suspended in 3 mL of distilled water, boiled for 15 min, and then acidified with 2 mL of 9.2 mol/L perchloric acid for 15 min. The supernatants were recovered through centrifugation and subjected to the anthrone-sulfuric acid method for measuring the starch concentration [18,19]. The non-structural carbohydrate concentration was the sum of the total soluble sugar and starch concentrations.

#### 2.3.4. Vitamin C Determination

Vitamin C from samples was determined as follows: a phosphomolybdic heteropoly acid solution was prepared by mixing 300 mL of 12% H_2_SO_4_, 50 mL of 5% ammonium molybdate, and 50 mL of 0.5% NaH_2_PO_4_, then heated to boiling and cooled. For the calibration curve, 2.5 mL of this solution was added to each of several 25 mL volumetric flasks, brought to slight boiling in a water bath, then immediately mixed with 0.0–0.30 mL of 1 mg/mL vitamin C standard solution, heated in boiling water for 10 min, cooled, diluted to 25 mL with 9% H_2_SO_4_, and measured at 820 nm. For sample analysis, 5 g of homogenized sample (accurately weighed to 0.0001 g) was extracted with 40 mL of 1% oxalic acid, followed by 0.25 mL of 30% ZnSO_4_ and 0.25 mL of 15% K_4_Fe(CN)_6_, then diluted to 50 mL with 1% oxalic acid. The filtrate was treated similarly to the standards. The vitamin C content (mg/100 g) was calculated as C × V × 100/(V_0_ × W), where C is the concentration (mg) from the standard curve, V is the total extract volume (50 mL), V_0_ is the aliquot volume taken for measurement, and W is the sample mass (g) [20].

#### 2.3.5. Lycopene Determination

Lycopene from tomato products was extracted as follows: 5 g of sample (tomato powder) was homogenized in 50 mL methanol plus 1 g calcium bicarbonate and 5 g of celite. The sample was then filtered through Whatman no.1 and no.42 filter papers. Lycopene was extracted using a sample of hexane: acetone (1:1, *v*/*v*), and quantified spectrophotometrically at 472 nm (UV-2600, Shimadzu Corporation, Kyoto, Japan) [21].

#### 2.3.6. Total Free Amino Acid Determination

The total free amino acid content was quantified by the Cadmium-ninhydrin reagent method [22]. Dissolve 1 g of calcium chloride (CaCl_2_) in 1 mL of water to obtain the cadmium solution. Next, measure 80 mL of absolute ethanol and 10 mL of acetic acid in a glass cylinder and mix gently. In a glass flask, dissolve 0.8 g of ninhydrin in the ethanol-acetic acid mixture using a magnetic stirrer. Once the powder is completely dissolved, add the previously prepared cadmium solution and mix thoroughly to obtain the working reaction solution. Prepare a series of calibration standard solutions using glycine at concentrations ranging from 0.2 to 20 mg/L for constructing a standard curve. The required equipment includes 1.5–2 mL conical tubes, a floating foam rack, and a thermostatic water bath. After the reaction is complete, terminate it using an ice bath. Absorbance measurements are performed using a spectrophotometer set at a wavelength of 507 nm (UV-2600, Shimadzu Corporation, Kyoto, Japan).

#### 2.3.7. RNA Extraction and RT-qPCR Analysis

Total RNA was extracted from pericarp samples using the Trizol reagent (Mei5bio Company Ltd., Beijing, China) following the manufacturer’s instructions. Subsequently, cDNA was synthesized by reverse transcription of 1 µg of total RNA using a cDNA synthesis kit (Vazyme Biotech Company Ltd., Nanjing, China). The RNA and complementary DNA (cDNA) concentrations were determined based on the A260 and A280 values using a NanoDrop ND-2000 photo spectrometer (Thermo Fisher Scientific, Inc., Waltham, MA, USA). Specific primers for quantitative reverse transcription PCR (RT-qPCR) were designed. Quantitative RT-qPCR was carried out with a final volume of 15 mL using SYBR qPCR Master Mix (Vazyme Biotech Company Ltd., Nanjing, China) and an iQ5 Multicolor Real-time PCR Detection System [23]. Melting curves were examined to detect unspecific amplifications and primer dimerization. For relative quantification, *SlACTIN* (Solyc03g078400) was used as an internal reference, and the 2^−^^△△CT^ method [24] was used. The threshold cycle (C_t_) value was normalized against *SlACTIN* and compared with the control samples. For gene expression analyses, relative expression values were log2-transformed.

### 2.4. Statistical Analysis

Statistical analysis was conducted using SPSS22.0 software (SPSS Inc., Chicago, IL, USA). For multiple comparison statistical tests, statistically significant differences among means were determined by one-way ANOVA with Duncan’s multiple range test at a significance of *p* < 0.05. Graphs were generated using GraphPad Prism 9 software (GraphPad Software, San Diego, CA, USA). The heatmaps were created by TBtools software (v1.09861; https://www.tbtools.com).

## 3. Results

### 3.1. Quality Traits of Red-Ripe Fruits Across Tomato Cultivars

The quality traits of red-ripe fruit serve as core indicators for evaluating tomato flavor, nutritional value, and marketability [25]. This study measured the TSS, total acidity, total free amino acids, vitamin C, and lycopene content in red-ripe fruit from eight different tomato cultivars.

**Figure 1:**
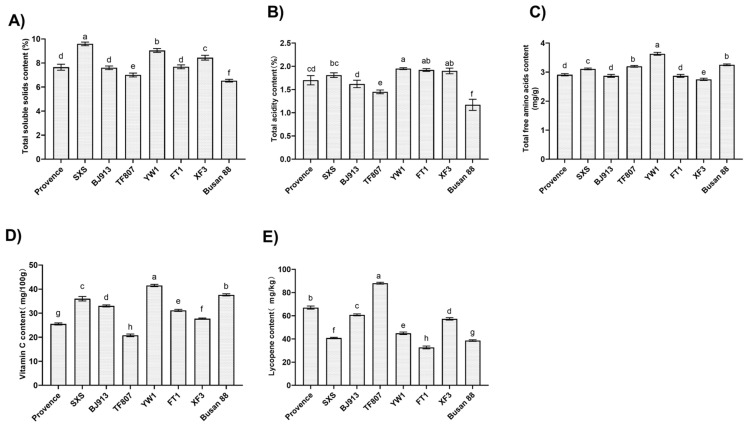
Fruit quality traits of different tomato varieties at the red-ripe stage. (**A**) Total soluble solids content, (**B**) total acidity content, (**C**) total free amino acids content, (**D**) vitamin C content, (**E**) Lycopene content. Data are presented as the mean ± standard deviation of three biological replicates (n = 3). Different letters indicate significant differences according to Duncan’s multiple range test (*p* < 0.05).

Significant variations in quality were observed among tomato cultivars at the red-ripe stage (Figure 1). TSS content was highest in ‘SXS’ (9.59%) and lowest in ‘Busan 88’ (6.53%) (Figure 1A). Total acidity content was highest in ‘YW1’ (1.95%) and lowest in ‘Busan 88’ (1.17%) (Figure 1B). Among nutritional components, ‘YW1’ exhibited the highest levels of both total free amino acids (3.63 mg/g) and vitamin C (41.51 mg/100 g), while ‘TF807’ showed the lowest vitamin C content (20.78 mg/100 g) (Figure 1C,D). Notably, the lycopene content in ‘TF807’ was significantly higher than that of the other cultivars, reaching 88.05 mg/kg, whereas ‘FT1’ had the lowest lycopene content (32.67 mg/kg) (Figure 1E). Overall, ‘YW1’ performed excellently in multiple nutritional components, ‘SXS’ showed superior sweetness, and ‘TF807’ demonstrated a significant advantage in lycopene. These cultivar-specific quality profiles provide valuable material for further investigating the mechanism underlying fruit quality formation.

### 3.2. Analysis of Carbohydrate Content and Starch Metabolism Gene Expression in Different Tomato Cultivars

#### 3.2.1. Variation in Carbohydrate Components Among Cultivars

The mature green stage represents a critical period for quality formation in tomato fruit, characterized by substantial accumulation of non-structural carbohydrates (NSCs) [5,26,27]. To compare NSC dynamics at this stage among different tomato cultivars, we quantified soluble sugars and starch—the principal components of NSCs—in eight representative cultivars.

**Figure 2:**
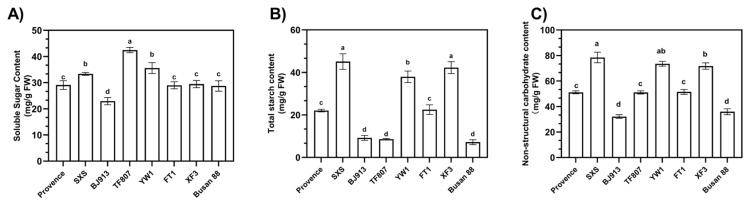
Variation in carbohydrate components among cultivars. (**A**) soluble sugar content, (**B**) total starch content, (**C**) non-structural carbohydrate content. Data are presented as the mean ± standard deviation of three biological replicates (n = 3). Different letters indicate significant differences according to Duncan’s multiple range test (*p* < 0.05).

Analysis of soluble sugar and starch content in mature green fruits of eight tomato cultivars revealed significant differences in both datasets (Figure 2A). The soluble sugar content in mature green fruits ranged from 22.91 mg/g FW to 42.45 mg/g FW among the eight cultivars. ‘TF807’ exhibited the highest soluble sugar content, reaching 42.45 mg/g FW, which was significantly greater than that of all other cultivars, indicating a strong capacity for sugar accumulation. ‘YW1’ (35.57 mg/g FW) and ‘SXS’ (33.35 mg/g FW) also showed relatively high soluble sugar levels, whereas ‘BJ913’ had the lowest content (22.91 mg/g FW).

The differences in starch content were even more pronounced, ranging from 7.20 mg/g FW to 45.09 mg/g FW. ‘SXS’ showed the highest starch content, while ‘Busan 88’ exhibited the lowest. Furthermore, fruits at the mature green stage of ‘XF3’ (42.27 mg/g FW) and ‘YW1’ (37.94 mg/g FW) also demonstrated relatively high starch levels. In contrast, ‘Busan 88’ (7.20 mg/g FW), ‘BJ913’ (9.20 mg/g FW), and ‘TF807’ (8.58 mg/g FW) were characterized as low-starch accumulation types (Figure 2B).

Based on the combined levels of soluble sugars and starch, the NSC contents of mature green fruits were further compared among cultivars. The results showed significant differences in NSC accumulation across the eight tomato cultivars (Figure 2C). ‘SXS’ and ‘YW1’ exhibited relatively high NSC contents, whereas ‘BJ913’ and ‘Busan 88’ showed comparatively low NSC levels. Overall, the variation pattern of NSC cultivars content was consistent with the combined trends of soluble sugar and starch accumulation.

#### 3.2.2. Analysis of Gene Expression Related to Starch Synthesis and Degradation in Tomato Fruits

To elucidate the molecular basis underlying cultivar-dependent differences in starch content and their effects on fruit quality at the red-ripe stage, the expression patterns of starch biosynthesis- and degradation-related genes were analyzed at the mature green stage. The results showed that significant differences existed among tomato cultivars in the expression levels of starch biosynthesis genes (*AGPS1*, *AGPL1*, and *AGPL2*) and starch degradation-related genes (*PWD*, *GWD*, and *BAM1*). Cultivars with higher starch content generally exhibited elevated expression levels of starch biosynthesis-related genes, whereas the expression of starch degradation-related genes was relatively lower. These findings provide a molecular explanation for the cultivar-dependent differences in starch accumulation.

**Figure 3:**
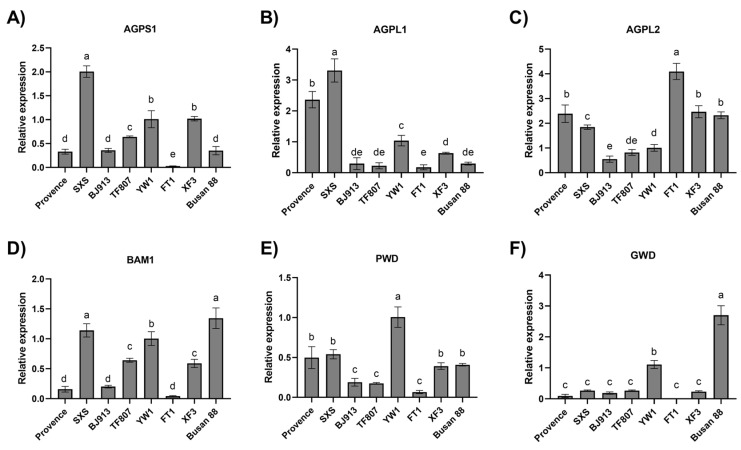
Expression of starch synthesis and degradation metabolism genes in tomato fruits at the mature green stage. (**A**–**C**) Expression of starch synthesis genes *AGPS1*, *AGPL1*, and *AGPL2,* (**D**–**F**) Expression of starch degradation genes *BAM1*, *PWD*, and *GWD*. Data are presented as means ± standard deviation of three biological replicates (n = 3). Different letters above bars indicate significant differences according to Duncan’s multiple range test (*p* < 0.05).

‘SXS’ and ‘XF3’ exhibited the highest starch contents (45.09 and 42.27 mg g^−1^ FW, respectively), which were significantly higher than those of the other cultivars. Correspondingly, gene expression analysis revealed that these two high-starch cultivars displayed similar transcriptional patterns at the mature green stage. The key genes involved in starch biosynthesis, *AGPS1* and *AGPL1*, were maintained at relatively high expression levels, whereas the expression of key genes initiating starch degradation remained comparatively low (Figure 3A,B). This coordinated gene expression pattern, characterized by enhanced biosynthesis and reduced degradation, collectively drove substantial starch accumulation in fruits at the mature green stage. In contrast, the low-starch cultivars ‘TF807’ and ‘Busan88’ exhibited an opposite transcriptional trend, with relatively lower expression levels of starch biosynthesis genes and earlier or more pronounced activation of certain starch degradation-related genes. This negative association further confirms that the balance between starch biosynthesis and degradation gene expression during the mature green stage constitutes a key molecular basis determining the final level of starch accumulation among tomato cultivars.

### 3.3. Correlation Analysis Between Carbohydrate Components, Starch Metabolism Gene Expression and Red-Ripe Fruit Quality

#### 3.3.1. Correlation Between Carbohydrate Components and Red-Ripe Fruit Quality

**Figure 4:**
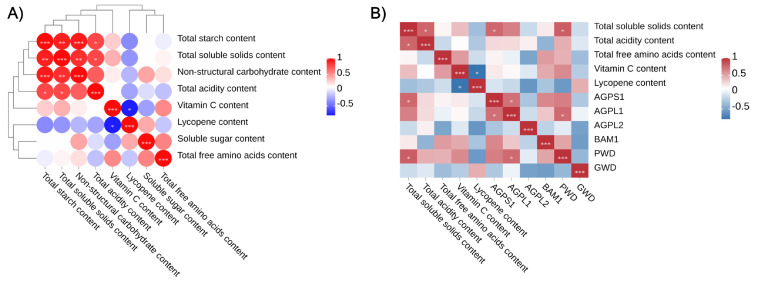
(**A**) Correlation heatmap between carbohydrate components and quality indicators at the red-ripe stage, (**B**) Correlation heatmap between starch metabolism gene expression at the mature green stage and quality indicators at the red-ripe stage. * indicates a significant correlation at the 0.05 level; ** indicates a highly significant correlation at the 0.01 level; *** indicates a highly significant correlation at the 0.001 level.

Correlation analysis was conducted between carbohydrate-related traits at the mature green stage and fruit quality attributes at the red-ripe stage (Figure 4A). The results revealed a highly significant correlation between starch content at the mature green stage and TSS content at the red-ripe stage (correlation coefficient = 0.922). The mature green starch content was also significantly correlated with total acidity at the red-ripe stage (correlation coefficient = 0.783). In addition, NSC content at the mature green stage showed a significant correlation with TSS at the red-ripe stage (correlation coefficient = 0.872). Among quality traits measured at the red-ripe stage, TSS content was significantly correlated with total acidity (correlation coefficient = 0.785).

#### 3.3.2. Correlation Between Starch Metabolism Gene Expression at the Mature Green Stage and Red-Ripe Fruit Quality

To investigate the relationship between the expression of starch synthesis- and degradation-related genes at the mature green stage and fruit quality traits at the red-ripe stage, correlation analysis was performed between the expression levels of three starch biosynthesis genes (*AGPS1*, *AGPL1*, and *AGPL2*) and three starch degradation genes (*BAM1*, *GWD*, and *PWD*) at the mature green stage and fruit quality parameters at the red-ripe stage (Figure 4B). The results showed that TSS content at the red-ripe stage was significantly and positively correlated with the expression levels of *AGPS1* and *PWD*, with correlation coefficients of 0.77 and 0.81, respectively. These results indicate that higher expression levels of the starch biosynthesis gene *AGPS1* and the starch degradation gene *PWD* at the mature green stage are closely associated with the final fruit flavor. In contrast, no consistent significant correlations were detected between the other starch synthesis- and degradation-related genes and additional fruit quality traits, including total acidity, total free amino acid content, vitamin C content, and lycopene content.

#### 3.3.3. Integrated PCA of Carbohydrate Metabolism, Gene Expression and Fruit Quality Traits

**Figure 5:**
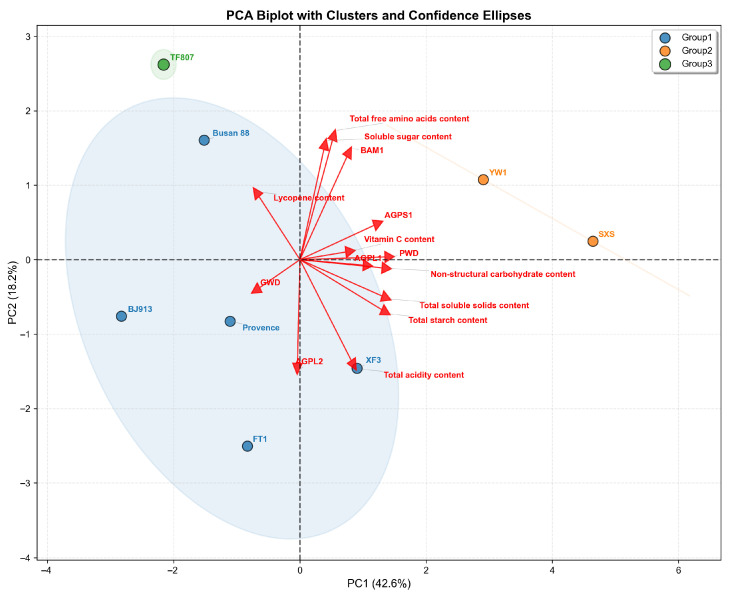
Principal component analysis (PCA) illustrating the relationships among carbohydrate-related traits and fruit quality parameters in eight tomato cultivars. The blue ellipse represents the 95% confidence ellipse for the Group 1 samples, illustrating their cluster distribution. The orange line connects the two Group 2 samples, highlighting their separation from the other groups along the PC1 axis.

An integrated principal component analysis (PCA) biplot was generated to examine the overall relationships among carbohydrate-related traits in mature green fruits, final fruit quality parameters across the eight tomato cultivars (Figure 5). The first two principal components explained 60.8% of the total variance (PC1 = 42.6%, PC2 = 18.2%). Clear separation among the cultivars was observed, and they were naturally grouped into three distinct clusters (Group 1, Group 2, Group 3) as indicated by confidence ellipses. PC1 served as the primary axis of separation and was strongly associated with key quality and metabolic traits. Variables such as total starch content, TSS, soluble sugar content, and the expression of the *AGPS1* gene were positively correlated with PC1. Consequently, cultivars located on the positive side of PC1, specifically ‘YW1’ and ‘SXS’ (Group 2, orange), were characterized by higher levels of starch, sugars, and soluble solids. In contrast, ‘TF807’ (Group 3, green) was positioned on the negative side of PC1, reflecting a distinct metabolic profile with lower values for these PC1-associated traits. The remaining five cultivars (‘Busan 88’, ‘BJ913’, ‘Provence’, ‘FT1’, ‘XF3’, forming Group 1, blue) were distributed around the central region of the biplot, indicating intermediate values for these major traits. Total free amino acids content, total acidity, and lycopene content showed a stronger association with PC2. The separation along PC2 highlights differences in flavor-related compounds (acids, amino acids) and nutritional components (lycopene) that are independent of the primary starch-and-sugar driven separation along PC1. Overall, the PCA results visually highlighted clear cultivar differentiation and supported the association patterns revealed by correlation analyses.

## 4. Discussion

TSS constitute a primary quality metric in tomato fruit, predominantly composed of soluble sugars, organic acids, and other nutritional constituents [12,14]. At the mature green stage, starch represents the main form of carbohydrate accumulation in tomato fruits, providing a critical carbon source for normal growth and the biosynthesis of flavor compounds [26,27]. Studies have indicated that in bananas, the total soluble sugar content increases from 1.8% to 18.6% from the early developmental stage to fruit ripening, concurrent with a decline in starch content during maturation [28]. Burdon et al. [29] found that the accumulation of starch in the early to middle stages of kiwifruit development establishes a carbon source foundation for the subsequent rapid increase in TSS. Furthermore, Valverde-Miranda, et al. [30] demonstrated a stable linear relationship between TSS and Dry Matter Content (DMC) in cucumber fruit. Given that starch constitutes a core component of DMC during the early and middle developmental phases, its accumulation level directly influences both the initial accumulation and the rate of TSS accumulation in the fruit. Therefore, elucidating the relationship between starch accumulation at the mature green stage and TSS at maturity holds important theoretical and practical significance for improving tomato fruit quality.

In this study, we quantified the contents of soluble sugar and starch in mature green fruits of eight tomato cultivars, as well as the compounds in red-ripe fruits, including TSS, total acidity, vitamin C, lycopene, and free amino acids. We further analyzed the expression of starch biosynthesis genes (*AGPS1*, *AGPL1*, *AGPL2*) and starch degradation genes (*BAM1*, *PWD*, *GWD*) in mature green fruits, and their correlation with quality indices of red-ripe fruits. The results showed that starch content in mature green fruits was highly positively correlated with TSS in red-ripe fruits (r = 0.922), whereas soluble sugar content showed no correlation with TSS. This indicates that starch accumulation during the mature green stage is a key factor influencing TSS at red-ripe stage, consistent with the findings of Osorio, et al. [31]. During the mature green stage, starch serves as the primary carbohydrate reserve, providing an essential carbon source for fruit growth and the formation of flavor compounds. During red ripening, starch is gradually degraded into glucose, fructose, and other soluble sugars, a process that contributes to the increase in TSS [32,33,34].

Further supporting this pattern, the varietal differences in starch accumulation were consistent with their final TSS content at ripening. High starch-accumulating cultivars such as ‘SXS’ and ‘YW1’ exhibited higher TSS content at the red-ripe stage. In contrast, low starch-accumulating cultivars like ‘TF807’, despite having relatively higher soluble sugar content at the mature green stage, showed comparatively lower TSS content upon full ripening. This suggests that in tomato quality improvement, relying solely on the accumulation of soluble sugars at the mature green stage may not significantly enhance final sweetness. Increasing starch reserves appears to be a more effective strategy. This finding aligns with the study by Tadesse, et al. [35], who indicated that the starch accumulation level during the expansion stage is a core determinant of final sugar content, whereas the influence of early sugar accumulation is limited.

The gene expression analysis further supports the above conclusions at the molecular level. Starch synthesis-related genes such as *AGPS1*, *AGPL1*, and *AGPL2* exhibited significantly higher expression in high starch-accumulating cultivars. Among these, the expression of *AGPS1* showed a correlation coefficient of 0.72 with starch content during the mature green stage, and was significantly positively correlated with TSS content at the red-ripe stage (r = 0.77). *AGPS1* is a key subunit of ADP-glucose pyrophosphorylase (AGPase), the rate-limiting enzyme in starch synthesis, and its fundamental role has been characterized in tomato and other species [36,37]. On other hand, starch degradation gene expression at mature green stage also affected fruit quality. *PWD*, which positively correlated with TSS content at the red-ripe stage (r = 0.81), is a recognized component of the starch degradation pathway, and its involvement has been directly identified in tomato [38]. Similarly, Fujita, et al. [39] demonstrated in rice that efficient starch degradation greatly increases sugar content at the red-ripe stage, which aligns with our finding.

This study also revealed that starch content at the mature green stage exhibited a significant positive correlation with total acidity at the red-ripe stage (r = 0.783), suggesting that starch accumulation may not only affect sweetness but also co-shape fruit flavor by modulating organic acid metabolism. The sugar–acid balance is a critical component of tomato sensory quality, with a higher sugar–acid ratio generally associated with better flavor perception [40]. Therefore, in breeding practices, it is essential to comprehensively consider starch accumulation, sugar conversion efficiency, and acid metabolism characteristics to achieve coordinated improvement in both flavor and nutritional quality [41,42,43].

Beyond the positive correlation between starch and TSS or total acidity, we observed a negative correlation between starch content at the mature green stage and lycopene content at the red-ripe stage. This suggests a metabolic trade-off between primary carbon storage and secondary carotenoid biosynthesis. The underlying mechanism may involve competition for common carbon precursors, as starch synthesis potentially diverts carbon flux away from the MEP pathway required for lycopene production, a notion supported by studies in starch-deficient mutants [44,45]. In contrast to TSS and total acidity, starch content in mature green fruits showed no significant correlation with vitamin C and free amino acid contents in red-ripe fruits. The biosynthesis of vitamin C (ascorbic acid) is closely associated with mitochondrial metabolism, cellular redox homeostasis, and developmental and environmental signals, rather than being directly regulated by carbohydrate storage levels [46,47]. Free amino acids mainly exist in the form of proteins or structural components, and their accumulation largely reflects nitrogen metabolism rather than carbohydrate storage and conversion processes [48]. It has been reported that protein and bound amino acid accumulation in fruit is primarily determined by nitrogen supply, amino acid assimilation capacity, and the rate of protein synthesis, and shows limited direct linkage with transient carbon reserves [48,49,50].

The integrated PCA provided a complementary overview of cultivar variation and trait associations, which was consistent with the correlation analyses performed in this study. The coordinated distribution of starch content, quality-related traits and several starch metabolism-related genes along PC1 supports the close association between early carbohydrate status and final fruit quality among different tomato cultivars.

## 5. Conclusions

In summary, this study demonstrates that starch accumulation in tomato fruits at the mature green stage plays a pivotal role in determining final fruit quality, as it shows a strong positive correlation with both total soluble solids (TSS, r = 0.922) and total acidity (r = 0.783) in red-ripe fruits, thereby co-shaping the critical sugar-acid balance. At the molecular level, the expression of the starch synthesis gene *AGPS1* and the degradation gene *PWD* is positively correlated with final TSS, providing a genetic basis for this relationship. While starch content shows no significant link to vitamin C or free amino acids, its negative correlation with lycopene suggests a metabolic trade-off. Collectively, these findings establish that modulating starch metabolism prior to ripening is a key strategy for breeding tomatoes with superior flavor.

## Data Availability

Data are contained within the article and supplementary materials.

## Supplementary Materials

The following supporting information can be downloaded at: https://www.mdpi.com/article/10.3390/plants15091364/s1. Table S1. Primer information of related genes; Table S2. 2023 Spring season; Table S3. 2023 Autumn season; Table S4. 2024 Autumn season.
