# Supplementary material for: Role of Starch Accumulation at the Mature Green Stage in Shaping Tomato Fruit Quality
Source: Plants (Basel). 2026 Apr 29;15(9):1364. doi: 10.3390/plants15091364 (PMC13164892; doi:10.3390/plants15091364)
Supplement: Supplementary file 1 [file plants-15-01364-s001.zip › plants-4239105-supplementary.pdf]

Supplement Table S1. Primer information of related genes

Genes	Gene ID	Primers sequence（5'-3'）	
SlAGPL1	Solyc01g109790	Forward	ATTATTGTTGGTGACTTTGTG
		Reverse	GACCTTTGGAAACCTATTGG
SlAGPL2	Solyc07g019440	Forward	AGCAGACTACTACCAAACAG
		Reverse	ATTCCAATCGGTACTTTCC
SlAGPS1	Solyc07g056140	Forward	TGTAAGATTCACCATTCCGT
		Reverse	TCTTCTATAATTGCTCCCTCTG
SlBAM1	Solyc09g091030	Forward	GTTCCACTTGCTGGGGAGAA
		Reverse	TGTTCGCGGCATTTGTTAGC
SlPWD	Solyc09g098040	Forward	AGGCAGTTGTTCCTGACGTA
		Reverse	CCTGATGTGTCCCACTTCCT
SlGWD	Sloyc05g005020.2.1	Forward	CAATGGGAAAGAAGCGTGCT
		Reverse	CTGAAGTGGGAGGCCTAACA
SlACTIN	Solyc03g078400	Forward	ATCCCAAGGCCAACAGAGAG
		Reverse	CGACCGCTAGCATACAGAGA

Supplement Table S2. 2023 Spring season

Cultivar	TSS (%)	Total acidity (%)
Provence	7.2	1.35
Sai Xishi (SXS)	9.3	1.58
Baoshijie 913 (BJ913)	6.7	1.18
Tiefan 807 (TF807)	6.9	1.22
Yuanwei No.1 (YW1)	8.2	1.88
Fentao No.1 (FT1)	7.5	1.38
Xiangfei No.3 (XF3)	8.0	1.46
Busan 88	6.9	1.15

Supplement Table S3. 2023 Autumn season

Cultivar	TSS (%)	Total acidity (%)
Provence	7.8	1.42
Sai Xishi (SXS)	8.6	1.65
Baoshijie 913 (BJ913)	7.1	1.24
Tiefan 807 (TF807)	7.2	1.28
Yuanwei No.1 (YW1)	8.7	2.01
Fentao No.1 (FT1)	7.9	1.43
Xiangfei No.3 (XF3)	8.5	1.53
Busan 88	6.9	1.20

Supplement Table S4. 2024 Autumn season

Cultivar	TSS (%)	Total acidity (%)
Provence	6.9	1.30
Sai Xishi (SXS)	8.4	1.55
Baoshijie 913 (BJ913)	6.5	1.15
Tiefan 807 (TF807)	6.6	1.18
Yuanwei No.1 (YW1)	8.4	1.91
Fentao No.1 (FT1)	7.6	1.36
Xiangfei No.3 (XF3)	8.1	1.48
Busan 88	7.1	1.12
